# Comprehensive cardiometabolic rehabilitation program for childhood obesity

**DOI:** 10.1186/s41043-025-01191-9

**Published:** 2026-01-03

**Authors:** Nicole González Concha, Litsbett Velásquez Fuentes, Denys Urrea Pereira, Jorge Díaz Olivares, Hugo Berríos-Arvey

**Affiliations:** 1 Estudiante del Programa de Magister en Dirección y Gestión Estratégica en Salud, Facultad de Economía, Negocios y Gobierno, Universidad San Sebastián, Santiago, Chile; 2 Departamento de Salud Pública, Facultad de Medicina, Universidad San Sebastián, Sede Valdivia, Chile. General Lagos 1163, Valdivia, Los Ríos Chile

**Keywords:** Obesity, Preventive medicine, Cost-effectiveness, Comprehensive health, Economy

## Abstract

**Background:**

Obesity is a public health problem in Chile, with 31% of adults and 54% of children affected. Current public policies present gaps in prevention with 44% of obese children. In Penco-Lirquén, a comprehensive and personalized Pediatric Cardiometabolic Rehabilitation program is proposed.

**Objective:**

Analyze the cost-effectiveness of program.

**Method:**

Characterization of supply and demand, together with a technical-financial study to determine viability and cost-effectiveness.

**Results:**

The economic viability with a positive Net Present Value and an Internal Rate of Return of 13% estimates that the interventions could be 49% cheaper than the current strategy and improve the Human Development Index by 0.585%.

**Conclusion:**

The implementation of the program is proposed as a cost-effective strategy, promoting healthy habits and optimizing health resources. The prevention and treatment of obesity must be prioritized globally to protect the health of future generations and reduce costs.

## Background

Globally, obesity is a growing health problem, with a high prevalence in adults and children. In Chile, about 31% of adults are obese and 54% of children are overweight or obese, which leads to the development of unfavorable physical, psychological and social conditions [1, 2]. Associated multimorbidity is a matter of interest in public health, with overweight being a factor that increases the risk of developing it by 26%, while obesity almost doubles it, which is why state policies present intersectoral strategies for its intervention [3]. 

In terms of preventive medicine, the Child Health Control strategy aimed at children from 0 to 9 years old, allows for the evaluation of the environment, identification of risks and enhancement of parenting skills, including comprehensive development, biopsychosocial approach, family environment, nutritional condition and diagnosis of health problems [4]. For its part, the Comprehensive Health Control of Adolescents, aimed at adolescents from 10 to 19 years old, focuses on the evaluation of their health and development status through the early identification of protective and risk factors, in addition to the provision of integrated services and continuity of care [5]. Finally, the Choose Healthy Life Program focuses on preventing and reducing chronic non-communicable diseases and promoting healthy habits, considering specific criteria such as overweight and obesity, to provide effective treatments with a multidisciplinary team. In addition, the Preventive Medical Examination (PME) is a periodic, voluntary health evaluation free of charge for the user, [6] Aimed at beneficiaries of the National Health Fund and Health Insurance Institutions, whose main objective is the early detection of prevalent diseases in the country, allowing control and treatment measures to be implemented immediately [7]. This corresponds to Current Health Technology (CHT).

In addition to preventive interventions, for the monitoring of users who already have pathologies associated with their metabolic control, there is the Cardiovascular Health Plan (CHP) in Primary Health Care (PHC), to reduce the incidence of cardiovascular events and prevent premature morbidity and mortality [8]. In addition, and specifically, Type 2 Diabetes Mellitus (DM2) is covered by the Explicit Health Guarantees (EHG) regime, with guaranteed access to diagnostic confirmation within 30 days of suspicion and treatments within 24 h after diagnostic confirmation [9]. Meanwhile, Arterial Hypertension (AHT) is also included in EHG for beneficiaries aged 15 years with diagnostic confirmation within a maximum of 45 days from suspicion and treatment within 24 h after confirmation; [10] Meanwhile, dyslipidemia (DLP) can be detected in asymptomatic phases of atherosclerotic cardiovascular diseases, so universal screening is recommended from the age of 20, including a fasting lipid profile when the screening is positive; although this condition is not EHG, its detection is included in the PME [11]. 

Despite these strategies, in Chile the 2023 National Health Survey reports that 45% of households have a member with a chronic disease, where 56.4% have conditions associated with obesity and cardiovascular diseases and the records of the 2017 National Health Survey refer to 17.5% of the population living with five or more diseases, causing the costs associated with obesity, both direct such as medical care, and indirect such as loss of productivity, to be of great interest [1, 2]. 

Evidence indicates that obese individuals face approximately 30% higher medical costs than those of normal weight, with an estimated 1.9% to 4.7% of total health care costs attributed to obesity [12]. However, there is no robust evidence regarding the social costs of childhood obesity since the associated diseases generally manifest in adulthood, which complicates the assessment. However, it has been reported that the lifetime costs for a 4-year-old child with obesity amount to €75,000, or CLP$82,502,250 [13]. Considering this, it is noteworthy that the Biobío Region shows an obesity prevalence of 35.5%, exceeding the national average of 31.4% [2].

Organizations such as the World Health Organization (WHO), the United Nations Development Program (UNDP) and the Organization for Economic Cooperation and Development (OECD) have worked to reduce obesity in several countries, but in Chile, despite strategies aligned with these objectives, the obesity rate continues to increase [14]. The OECD has noted that implementing new, effective strategies could generate a positive impact on public health and the economy, with significant savings in health expenditure. In Chile, 2.9% of total health expenditure is allocated to obesity, representing 0.8% of the Gross Domestic Product (GDP), suggesting the need for economic evaluations of health strategies, which play a crucial role in transforming the way in which medical challenges are faced and improving the diagnosis and treatment of diseases, aligned with the National Health Strategy to achieve the Health Objectives by 2030 [15]. 

In terms of known interventions, the Active Brains clinical trial demonstrated that a 20-week exercise program improved cardiometabolic health in children with overweight or obesity [16] and a study by the Autonomous University of Chile concluded that physical activity alone does not reduce the Body Mass Index (BMI) in Latin American children and adolescents, [17] suggesting the need to combine it with dietary interventions. On the other hand, a Cochrane review in 2003 showed that multi-component interventions are beneficial in reducing BMI in children aged 6 to 11 years, [18] and the Bright Bodies Program at the Yale Pediatric Obesity Clinic showed positive results in obese children after 12 months of follow-up, with significant changes in anthropometric and metabolic parameters [19]. 

The limitations of the TSA support the creation of a new intervention program with a multidisciplinary approach, led by specialized health professionals and designed in a comprehensive and personalized manner, based on the preferences and needs of children and adolescents.

## Research objective

Determine the cost-effectiveness of a Children’s Cardiometabolic Rehabilitation (CCMR) program applied in children and adolescents with obesity between 5 and 14 years of age in the commune of Penco-Lirquén.

### Method

The efficiency evaluation of an CCMR program for children and adolescents with obesity was developed, aimed at beneficiary users of the Institutional Care Model in Family Health Centers of PHC, in the commune of Penco-Lirquén, Biobío, as a New Health Technology (NHT). A cost-effectiveness economic evaluation design was developed, the sequence of which considers characterizing the demand, defining the supply, carrying out a technical and financial study of the proposal for a five-year projection and evaluating viability based on Net Present Value (NPV) and Internal Rate of Return (IRR). Subsequently, structure, result and process indicators were defined with projections of marginal productivity that allow comparing the cost-effectiveness of the contrasted proposal with the current strategy.

The cost analysis of the current strategy, both for children and adults, considering the expenses involved in the treatment of chronic pathologies derived from obesity, was carried out with data obtained through the transparency law of government organizations, detailing costs by type of service for the population of the National Health Fund or *Fondo Nacional de Salud (FONASA)* in Chile. The most relevant interventions for the diagnosis and treatment of chronic diseases directly related to obesity in adults were included.

## Demand characterization

The Penco-Lirquén commune, according to the 2017 census, has a population of 50,050 inhabitants, with 48.5% men and 51.5% women, and 99.0% urban. The poverty rate is 8.6% and childhood obesity affects 242 children between 5 and 14 years old. The health system includes the Penco and Lirquén Family Health Center (CESFAM), which in 2021 registered 387 admissions to the cardiovascular health program, representing an increase of 74.0% compared to the previous year. Table 1 shows the number of users with chronic pathologies associated with obesity.

**Table 1:** Users with pathology associated with obesity in Penco-Lirquén

Diagnosis	Total		
	Both sexes	Men	Women
HBP	7,689	2,994	4,695
DM2	3,684	1,390	2,294
DLP	3,842	1,358	2,484

Absolute population values ​​2021

There is a high prevalence of obesity in children, with coverage poorly targeted towards this population. There is also a high incidence of chronic pathologies in adults and little evidence on financial analyses available, due to a low urgency of the problem in the coverage objectives of health teams. In addition, there is a lack of participation and commitment of the target population, the community and health care networks in the use of health technologies aimed at this problem. The current health approach focuses mainly on the treatment of chronic pathologies, without prioritizing prevention or early approach to obesity.

Therefore, a gap is observed in the fulfillment of health objectives with attention to the comprehensive scorecard that regulates the family health and preventive medicine model. Table 2 shows the cost of the current strategy aimed at children and adolescents, while Table 3 shows the costs associated with the benefits demanded by adult users with pathologies attributable to obesity and Table 4 shows the costs associated with the common care demand of healthy adults.

**Table 2:** Cost of benefits provided to users with pathologies associated with obesity

Code	Benefit	Price ($)	Description
102,008	Nursing consultation	1,500	Assessment
101,309	Medical consultation	20,280	Initial, Middle, Final Consultation
102,010	Nutritional consultation/control Physical activity session	1,500 96,048	Session (1) Session (3 weekly)
102,010	Nutritional consultation/control	9,000	Evaluation (6 sessions)
903,002	Psychology consultation/control	7,340	Initial, Final Evaluation
	Paraclinical Examinations	26,720	Lipid Profile + Glycemic x2
Annual Total		162,388	Unit cost

Reference preces (CLP$) by MAI FONASA 2023 Coding

**Table 3:** Penco-Lirquén user demand

Disease	Type	Price ($)	Concentration	$ Unitary	Users	$ Annual
DM2	Diagnosis	9,400	1	9,400	387	3,637,800
DM2	Diagnosis	32,896	1	32,896	387	12,730,752
DM2	Treatment	68,081	1	68,081	3,684	250,810,404
HBP	Treatment	9,267	1	9,267	7,689	71,253,963
DLP	Diagnosis	1,370	2	2,740	3,842	10,527,080
DLP	Diagnosis	8,510	2	17,020	3,842	65,390,840
DLP	Diagnosis	6,280	2	12,560	3,842	48,255,520
DLP	Treatment	1,380	6	8,280	3,842	31,811,760
Annual total				160,244		494,418,119

Reference preces (CLP$) 2021

**Table 4:** Cost of benefits granted to users without pathology associated with obesity

Code	Benefit	Price ($)	Description
302,067	Total cholesterol test	2,740	2 exams a year
101,001	Medical consultation	20,280	3 checks a year
302,093	Glycemic test	2,680	2 exams a year
Annual total		25,700	Unit cost

Reference preces (CLP$) by MAI FONASA 2023 Coding

The above shows an important difference in the State’s costs when intervening in users who already have pathologies associated with obesity, compared to the costs of common care for healthy users; In addition, the costs associated with the needs of children and adolescent users who access a comprehensive care program when they have risk factors for childhood obesity are considered [20]. This unit cost is like the unit cost of the sick adult population; however, since chronic demand does not occur, the total costs are significantly lower in early intervention.

## Definition of the offer

The National Zero Obesity Strategy 2020, promoted by the Health’s Ministry, is based on the Five Circles of Healthy Living, a comprehensive approach that seeks to prevent and reverse obesity in the Chilean population.

These circles are:

1. Promotion of healthy eating with access to nutritious foods, policies such as food labeling, educational campaigns and regulation of marketing aimed at children.
2. Promotion of physical activity in daily life through community interventions, promotion of school sports and improvements in public infrastructure.
3. Mental health care by promoting stress management, self-care and emotional support programs in primary care networks.
4. Control of metabolic risk factors, promoting the timely diagnosis and management of metabolic conditions at the primary and secondary care levels.
5. Creating healthy environments that facilitate healthy choices, such as access to fresh food, safe environments for recreation and active transportation.

The five pillars work in a coordinated manner to address the social and structural determinants of obesity, with a special focus on the early stages of the life cycle. It is proposed that the offer considered include key elements that address these fundamental pillars of comprehensive well-being and act on the main modifiable determinants of obesity and its comorbidities.

- Healthy eating through nutritional benefits, with a positive impact on weight and metabolic health through a balanced diet that reduces the risk of obesity and chronic diseases. In addition, eating habits are formed at early stages of life, so promoting healthy eating from childhood helps prevent future problems [21].
- Physical activity, which tends to regulate weight and where exercise helps maintain an adequate energy balance, essential to prevent imbalances [22]. The systematic benefits of physical activity go beyond weight control, as it improves cardiovascular health, reduces the risk of several types of cancer and strengthens the musculoskeletal system. Given the increasing sedentary lifestyle associated with office work, the use of technology and motorized transport, promoting movement is more crucial than ever [23].
- Mental health, with its bidirectional relationship with obesity, since it can contribute to the development of psychological disorders such as anxiety and depression, while these, in turn, can lead to unhealthy behaviors such as overeating or a sedentary lifestyle. Strengthening self-care and emotional well-being is essential to adopting and maintaining lifestyle changes. Programs that promote emotional resilience and stress management increase adherence to healthy lifestyles. However, social and economic challenges, such as poverty or job insecurity, can negatively influence health-related decision-making [24].

These three elements act in synergy, affecting not only body weight, but also quality of life and the prevention of various diseases. An Internal and External Factors Evaluation Matrix of the current strategy was carried out to identify key points susceptible to improvement, as shown in Table 5. Success factors include the availability of multidisciplinary professionals and a comprehensive care program, while factors influencing failure are low urgency and weak financial analysis in technological innovation. The Matrix was constructed through a SWOT analysis, where the weights and ratings of each factor were determined through the consensus of the research team, ensuring a systematic weighting based on the Hanlon model.

**Table 5:** Factor evaluation matrix for the current intervention strategy in obesity

Matrix	Factor	Wight	Qualification	Weighing
External	Multidisciplinary teams that exist in the community	0.250	1	0.250
	Gap in the design and implementation of new health projects	0.075	2	0.150
	Problem of interest in WHO strategic guidelines	0.200	4	0.800
	Resources for new health intervention strategies	0.100	1	0.100
	Sustained increase in the prevalence of chronic diseases in the population	0.075	3	0.225
	Low sense of urgency of the problem within the coverage objectives	0.100	2	0.200
	Lack of participation and commitment of the target population	0.125	2	0.250
	Health approach aimed at treatment and not prevention	0.075	3	0.225
Internal	Existence of a health strategy and devices in the community	0.250	4	1.000
	Availability of updated literature regarding the problem under study	0.050	3	0.150
	Public policies between sectors with progressive development	0.125	4	0.500
	Increased social and political interest in quality of life of people	0.250	3	0.750
	High prevalence of children and adolescents with obesity in the commune	0.100	2	0.200
	Deficit in health technologies with a focus on the obese child population	0.075	1	0.075
	High prevalence of chronic pathologies in the adult population in the commune	0.100	1	0.100
	Weakness in financial analysis available with respect to the strategy	0.050	1	0.050

The matrix originates from an analysis of current strengths, opportunities, weaknesses and threats

Therefore, it is proposed to evaluate the strategy considering the characteristics of the demand and the purpose of delivering an intervention plan that includes accessible and attractive tools to address obesity in children and adolescents, through comprehensive services from health professionals, including general practitioners, kinesiologists, nutritionists and psychologists, who will focus on the personalized approach to physical activity, [25] nutrition and behavior, [17] adapting to the individual needs and preferences of each user [26]. 

## Feasibility study

The creation of a comprehensive program in the Penco-Lirquén commune is proposed, adapted to local needs and financed through Primary Care Reinforcement Programs. This program includes the implementation of a Comprehensive Treatment Center in a residential home that meets the necessary health requirements, focusing on children and adolescents from 5 to 14 years old and projected to 5 years according to recommendations of the Ministry of Health.

The technical study evaluated the necessary resources using data on the prevalence of childhood obesity in the region. Tangible and intangible assets essential for the operation of the center were identified, applying a Linear Depreciation method to estimate their value over 5 years and calculating the Commercial Residual Value of the fixed assets.

This feasibility study seeks to ensure the sustainability and efficiency of the center in the intervention of childhood obesity in the community. The analyses in Table 6 indicate a positive Net Present Value (NPV), which concludes that the project is economically viable, with an Internal Rate of Return (IRR) of 13%, thus recommending the execution of the project.

**Table 6:** Synthesis of the technical and financial study of the NHT proposal

Assets		Total Value ($)					Depreciation ($)			Amortization ($)	
Fixed asset		17,540,651					2,280,824			---	
Nominal asset		289,200					---			289,200	

**Item**				**Amounts**							
Investment ($)					17,540,651						
Fixed cost ($)					108.828,862						
Variable cost ($)					646,440^1^ 6,269,534^2^ 6,440,656^3^ 6,436,402^4^ 6,527,034^5^						
Annual income ($)					106,496,940						
Expenses ($)					611,990						
Working capital ($)					215,480^0^ 226,900^1^ 238,926^2^ 251,589^3^ 264,923^4^						
Accumulated depreciation ($)					11,404,120						
Book value ($)					6,136,531						
Scrap value commercial ($)					14,461,539						

**Period**	**T** ^**0**^		**T** ^**1**^			**T ** ^2^		**T** ^**3**^	**T** ^**4**^	**T** ^**5**^	
Flow ($)	−18,260,811		−1,435,608			−1,420,705		2,792,484	7,336,311		26,820,787
NPV					$3,022,387						
IRR					13%						

Superscript indicates the value applied to period T of the TSN project application

## Organizational study

The organizational study focused on defining the team of professionals that will make up the Comprehensive Treatment Center, determining its administrative structure and the work plans necessary for its operation. It is considered for this:

- Fixed Cost: Remunerations, basic services, maintenance and supplies.
- Variable Cost: Training, replacement coverage and extra-programmatic activities.
- Income: Comes from the benefits included in the CCRM basket.
- Expenses: Related to the operation and production of services in the health center.
- Working Capital: Initially CLP$587,545 was calculated, with reinvestment projections.
- Pure Flow: Projection of income and expenses generated by the project over 5 years.
- Feasibility: The NPV is positive, and the IRR is estimated at 13%.

Finally, to identify the efficiency of the comprehensive RCMI program, the costs of the CHT and NHT were analyzed, adjusted to the characterization of the users of the commune of Penco-Lirquén. In addition, structure, process and result indicators were built with the objective of determining the effectiveness of the proposal presented.

**Fig. 1:**
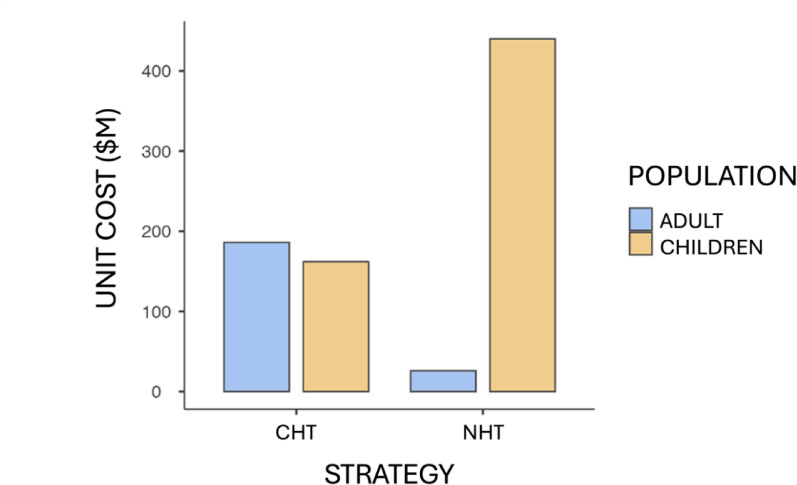
Unit costs according to healthcare technology in CLP$. Unit costs ($M = Thousands of Chilean pesos) for the adult population decrease while those for the child population increase when incorporating the new strategy proposed as a preventive intervention, which subsequently translates into long-term savings by avoiding chronic diseases in adulthood

According to the evidence collected, it is estimated that 44% of BGA with obesity will be obese adults prone to developing DM2, HBP and DLP; while the remaining 56% will eventually become a healthy adult whose care basket, unlike the obese group, will only incorporate PME. The health cost attributable to CHT and NHT available for both children and adults is shown in Table 7. The above can be analyzed considering the projection of the 44% probability of being an obese adult, representing the unit costs associated with each health technology, in adults and children, as shown in Fig. 1.

**Table 7:** Unit costs associated with healthcare demand for each strategy

		CHT	NHT
Adult	Disease Cost ($)	160,244	---
	PME Cost ($)	25,700	25,700
BGA	Total cost	162,388	440,070

There is no cost associated with adults on NRT since the pathological condition is prevented

**Fig. 2:**
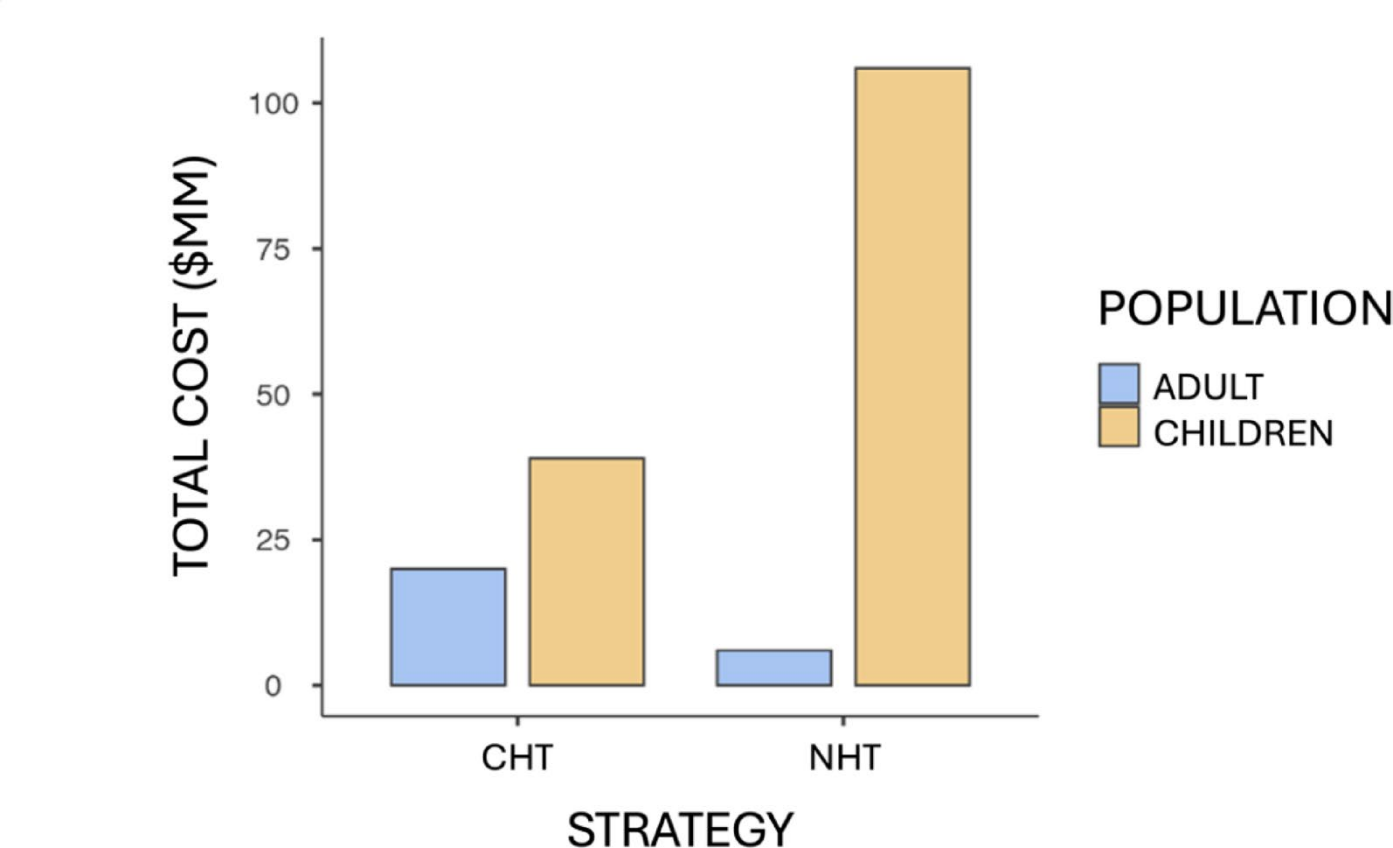
Total costs according to health technology in CLP$. Total costs ($MM = Millions of Chilean pesos) for the adult population decrease while those for the child population increase when incorporating the new strategy proposed as a preventive intervention, which implies long-term savings by avoiding chronic diseases in adulthood

In the commune of Penco-Lirquén the total number of children and adolescents diagnosed with obesity corresponds to 242 users, therefore, it is projected that 106 of them will become obese adults. When applying the costs to this population, the values ​​are shown in Table 8 and represented in Fig. 2.

**Table 8:** Total costs associated with healthcare demand for each strategy

		CHT			NHT		
		$ Unitary	Users	$ Total	$ Unitary	Users	$ Total
Adult	Disease	160,244	106	16,985,854	---	---	---
	PME	25,700	136	3,495,200	25,700	242	6,219,400
BGA	CCMR	162,388	242	39,297,896	440,070	242	106,496,940
Total ($)		59,778,960					

There is no cost associated with adults on NRT since the pathological condition is prevented

Given the above, it is suggested that NHT has a higher cost than CHT, however it is necessary to relate other epidemiological data of the commune that guides regarding the impact of these costs sustained over time. In this sense, in the commune of Penco-Lirquén the largest expenditure for the treatment of chronic pathologies occurs when intervening on users with DM2, reaching an estimated value of CLP$267,178,956 annually. In addition to this, this disease constitutes a history that increases cardiovascular risk and is associated with multiple complications, significantly affecting the quality of life of those who suffer from it [27, 28].

### Project indicators

The project included a series of indicators to evaluate the efficiency and impact of health technologies in the treatment of metabolic conditions and human development in the Penco-Lirquén commune.

#### Regarding the cost relationship of health technologies

**Fig. 3:**
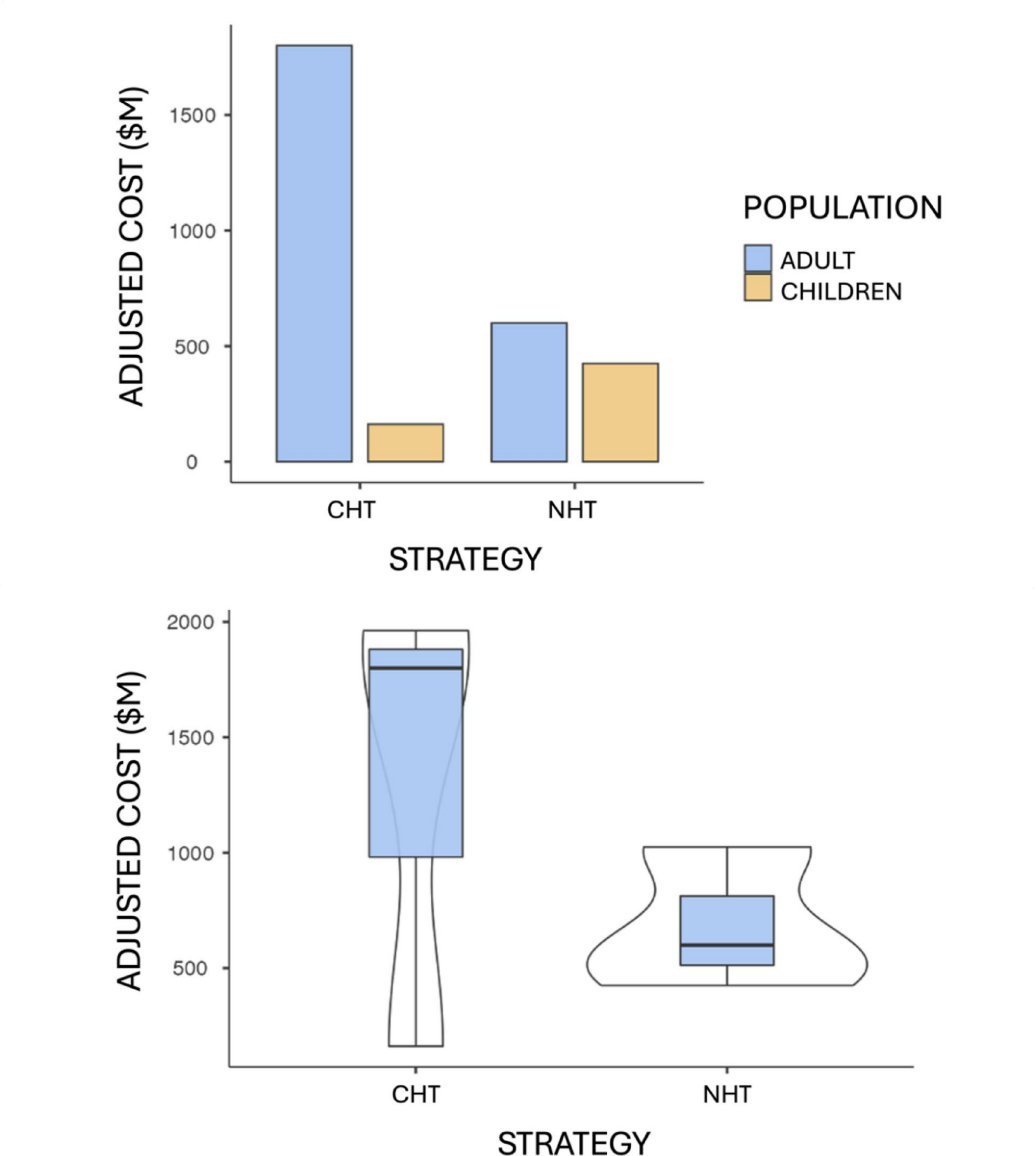
Unit costs adjusted according to health technology in CLP$. When making cost adjustments for the productive capacity and life expectancy of the sick population, the cost ratio changes, being significantly lower in the adult population operated on with the new proposal. Additionally, although the costs for intervention in the child population are higher with the new proposal, the concentration of global costs is significantly lower with the new strategy. $M represents thousands of Chilean pesos

Life expectancy in Chile, for people diagnosed with DM2 on average at age 47, is 79.5 years. Patients with DM2 have a life expectancy of 26.5 years after diagnosis, compared to 32.5 years for those without diabetes. When adjusting the costs of each TS according to life expectancy, it was found that NHT has a cost of 0.51 times that of CHT, indicating significant savings, as represented in Fig. 3, serving as a structure indicator.

#### Percentage of variation in the human development index (HDI)

**Fig. 4:**
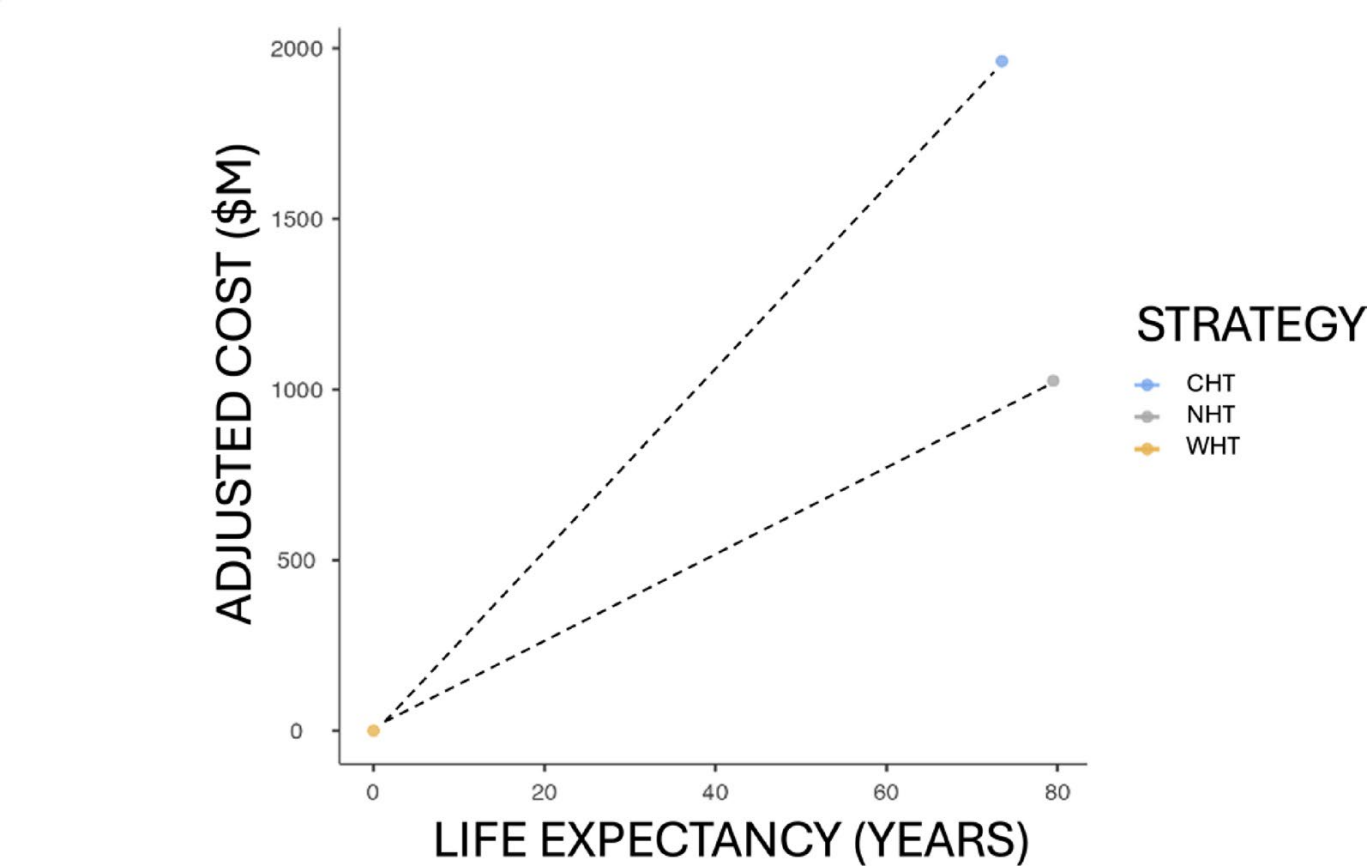
Projection of cost-effectiveness between both strategies. The cost-effectiveness analysis shows that, from the beginning without intervention on the sick population (WHT), CHT has almost double the cost than NHT, while achieving a lower life expectancy as an effect. The graph shows that NHT is more cost-effective than CHT

The HDI, which evaluates health, education and standard of living, allows development in countries to be measured in a global comparison. Given that the effects of metabolic diseases associated with obesity decrease life expectancy, the application of NHT compared to CHT shows an increase of 0.585% in the HDI, evidencing an improvement in human development when implementing the new technology, serving as a result indicator as detailed in Table 9 and represented in Fig. 4.

**Table 9:** Calculation of HDI attributable to the intervened population for both strategies

Variables	CHT	NHT
Life expectancy	78.9	79.6
Expected years of schooling	16.7	16.7
Average years of schooling	10.9	10.9
GDP per capita PPP	24,563	24,563
HDI	0.855	0.860

The variation in schooling and productivity attributable to obesity has not been considered

#### Percentage of referral of users to the program

This indicator is designed to evaluate the inclusion of users in the RRCM program; however, it has not yet been able to be calculated because the program has not been implemented. It is recommended to perform this calculation monthly once the program is in operation to record the derivations and propose improvements, so that it can be used as a process indicator.

These indicators will allow us to measure the impact of the Comprehensive Center on the health of the child and adult population, as well as its contribution to human development in the region. The details of the construction of the indicators are presented in Table 10.

**Table 10:** Proposed indicators for the evaluation of the strategy

Indicator title	HT cost ratio
Purpose	Determine the cost ratio achieved by projecting the years of life of users with DM2 in relation to the population without this morbidity
Type	Structure
Formula	$\frac{NHT\ Cost}{CHT\ Cost}$
Data source	National Health Survey – Local Statistical Registry
Threshold	>0
Periodicity	Annual
Methodology	Random sampling
Responsible	Director – Health Service Reference
Observations	---

Indicator title	Percentage of HDI variation
Purpose	Compare the HDI without and with CCMR based on life expectancy due to early risk factor intervention
Type	Result
Formula	$\frac{(HDI\ NHT - HDI\ CHT)\times 100}{HDI\ CHT}$
Data source	National Health Survey - Central Bank
Threshold	>0%
Periodicity	Annual
Methodology	Random sampling
Responsible	Director – Health Service Reference
Observations	---

Indicator title	Referral ratio of users entering NHT
Purpose	Measure user referrals from PHC according to the criteria established in the program
Type	Process
Formula	$\frac{BGA\ Number\ with\ entry\ criteria\ reffered\ to\ NHT}{Total\ Number\ BGA\ with\ admission\ criteria\ attended\ to\ in\ PHC}$
Data source	Statistics Department of the Ministry of Health
Threshold	0,8
Periodicity	Monthly
Methodology	Population
Responsible	Liaison Manager
Observations	---

## Discussion

The results obtained in this research underscore the urgent need to address childhood obesity in Chile, whose prevalence exceeds the OECD average. The limited effectiveness of current health technologies, evidenced by an 8% adherence rate in 2022, highlights the demand for more personalized and preventative approaches. In this context, the intervention strategy based on New Health Technologies (NHT) emerges as an innovative proposal, with a comprehensive approach that incorporates the active participation of health professionals.

The economic viability of NHT, reflected in a positive Net Present Value and an Internal Rate of Return greater than 10%, justifies its implementation despite a higher initial cost. This model demonstrates that investment in prevention and personalization generates long-term economic and social benefits, such as a reduction in comorbidities, increased life expectancy, and a favorable impact on the Human Development Index (HDI). NHT differentiates itself from Legacy Health Technologies (LHT) by offering personalized sessions, complementary activities—such as workshops and excursions—and the active inclusion of the family in the cardiometabolic rehabilitation process, with the collaboration of a multidisciplinary team composed of physicians, physical therapists, nutritionists, and psychologists.

The projected impact of the NHT on the HDI and life expectancy highlights its transformative potential for public health, positioning Chile as a regional benchmark. However, to maximize these benefits, it is essential to ensure the strategy’s sustainability through robust public policies that ensure ongoing funding, professional training, and systematic monitoring of results.

### Limitations

Among the main limitations of the study is the nature of the cost data, as publicly available data were used, without considering possible variations in market costs. Furthermore, the results depend on certain assumptions used in the projections, such as the estimate that 44% of children with obesity will remain obese into adulthood. There could also be an underestimation of indirect costs, for example, the productivity losses of parents when accompanying their children to appointments. Furthermore, the findings have limited generalizability to other municipalities or regions with different socioeconomic characteristics.

The study is also limited by the lack of longitudinal and actual follow-up data, given that the program has not yet been implemented. Furthermore, possible variations in financing or operating costs, as well as qualitative aspects such as user adherence or satisfaction, which could influence the program’s effectiveness, were not considered.

## Conclusion

The economic evaluation of early intervention for childhood obesity in the Penco-Lirquén commune demonstrates the importance of urgently addressing this problem in Chile. The results confirm that implementing NRT is economically viable and has the potential to significantly improve the human development and life expectancy of the affected population.

The cost-effectiveness ratio, with NHT being superior to CHT, indicates that adopting NHT represents an efficient strategy for optimizing health system resources. Furthermore, the implementation of NHT has demonstrated a 0.585% increase in the HDI, reflecting improvements in health, education, and living standards indicators. This demonstrates that the fight against childhood obesity transcends the health sector to positively impact regional social and economic development.

The NHT-based strategy, which integrates the collaboration of physical therapists, nutritionists, and psychologists, emphasizes the importance of a comprehensive and personalized approach. This model not only focuses on treatment but also involves families and promotes sustainable behavioral changes, key factors for long-term effectiveness.

To optimize results, it is essential to ensure the sustainability of the model through public policies that guarantee ongoing funding, adequate professional training, and an effective monitoring system. Collaboration between the public and private sectors can strengthen this strategy and facilitate its successful implementation.

Finally, we recommend that future research evaluate the adaptability of NHT in various contexts and populations, as well as the relationship between family interventions and long-term adherence. These studies will allow for the strategy to be adjusted and strengthened, expanding its impact on public health both nationally and internationally.

## Data Availability

The data analyzed in this document correspond to a technical and financial study attached in the appendices of this article. On the other hand, the prices of the services are extracted from the National Health Fund for the Institutional Care Model, as detailed in the tables of the financial study. Other data can be requested from the corresponding author.
